# An ABC transporter as a potential target against SHH-Medulloblastoma: From Benchtop to Bedside

**DOI:** 10.18632/oncotarget.28272

**Published:** 2022-09-08

**Authors:** Jingwen Zhu, Joseph N. Miller, John D. Schuetz

**Keywords:** transporter, chemotherapeutics

**Figure 1:**
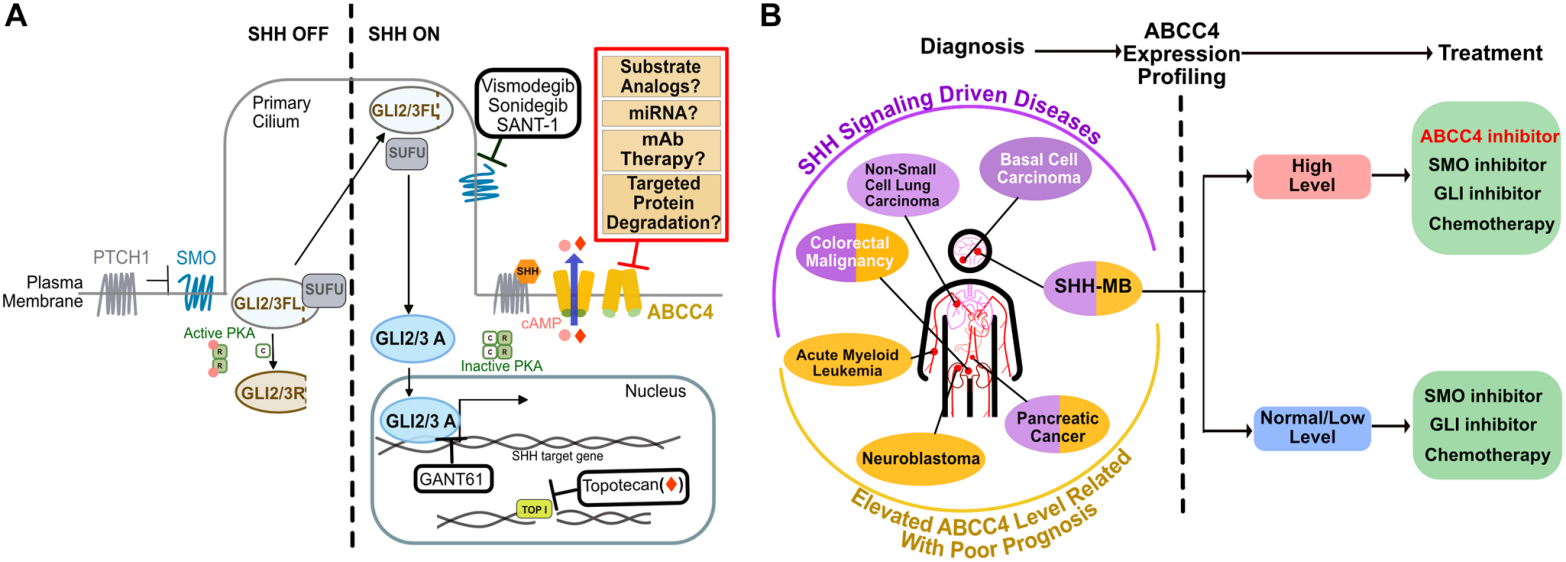
ABCC4 inhibition as a potential therapeutic strategy against SHH-MB. (**A**) A diagram of the SHH signaling pathway with its current inhibitors and therapeutics against SHH-MB (black boxes), and potential strategies to inhibit ABCC4 (red box). (**B**) A scheme depicting how to achieve efficacy of an ABCC4 inhibitor. After diagnosis, ABCC4 expression profiling would be recommended to ensure that the treatment of SHH-MB with an ABCC4 inhibitor only targets those patients with elevated ABCC4 level. This strategy may be applicable to other cancers arising from dysregulated SHH signaling (purple ellipses) or other malignancies in which high ABCC4 expression is a hallmark of poor prognosis (yellow ellipses). Among these diseases, black font indicates cancers in which ABCC4 has been suggested as a therapeutic target whereas white font indicates diseases in which ABCC4’s impact is still unclear.

Medulloblastoma (MB) is a common malignant pediatric brain tumor divided into four main subgroups (WNT, SHH, Group 3 and 4) [1]. The most prevalent MB in children <3 years is the Sonic Hedgehog (SHH) subtype, which arises from granule neuron progenitors with aberrant SHH signaling [1]. While a standard treatment regimen includes tumor resection followed by a combination of craniospinal irradiation and chemotherapy, recommended treatment for patients <3 years consists of only chemotherapeutics as radiation therapy produces neuro-developmental side-effects [2]. For SHH-MB, smoothened (SMO) inhibitors were initially seen as a promising therapy as SMO is central to SHH pathway activation (Figure 1A). However, this approach yielded diminishing returns for young children due to: 1) SMO acquired therapy-induced drug-resistant mutations [1]; 2) treatment with these inhibitors lead to irreversible developmental defects [3]; and 3) gene alterations in SHH signaling downstream from SMO circumvent SMO inhibition [1]. Thus, the identification of novel targetable regulators that function downstream of SMO is desirable to enhance SHH-MB therapy.

While the involvement of ATP-Binding Cassette (ABC) transporters in drug resistance is well studied, emerging studies have discovered their biological roles in tumorigenesis or cancer progression. One such study was published by Wijaya et al., who used a data-driven systems biology approach to reverse engineer a MB-specific interactome that identified unreported candidate drivers of SHH-MB [4]. Ultimately, the authors uncovered ABCC4, a C subfamily member of ABC transporters, as a likely modulator of the SHH pathway by demonstrating that ABCC4 transports membrane-residing cAMP, modulating PKA activity, a known negative regulator of SHH signaling [5] (Figure 1A). This mechanism highlights ABCC4’s role as a regulator downstream of SMO, shedding light on the potential application of ABCC4 inhibitors acting as novel single-agent therapeutics or in concert with traditional therapies against SHH-MB. While exciting, implementing these findings in the clinic may prove challenging.

First, further elucidation of the molecular mechanisms by which ABCC4 drives SHH-MB warrants additional investigation. Understanding ABCC4’s mechanism will guide the development of specific ABCC4 inhibitors. Assuming the authors’ discover that ABCC4’s regulatory role in SHH signaling depends on membrane-residing cAMP transport, inhibitory molecules may be designed as ABCC4 substrate-analogs to block ABCC4 transport via competitive inhibition, and in fact, previous reports have identified several small molecules working in this manner [6]. Unfortunately, many of these inhibitors were non-specific as they indiscriminately acted on other ABC family transporters and have only been used in pre-clinical investigations [6]. Recently, two ABCC4 selective inhibitors, Ceefourin 1 and Ceefourin 2, were generated, demonstrating greatly improved specificity towards ABCC4 and enhanced efficacy *in vitro* [7]. Another necessary characteristic of ABCC4 inhibitors as chemotherapeutics is tumor specificity. Because ABCC4 is expressed in a wide variety of tissues and impacts metabolism and pharmacokinetics [8], treatment with ABCC4 inhibitors that are not specific to the tumor could exacerbate chemotherapy-induced toxicity and/or fail to inhibit ABCC4 in the tumor. Thus, to generate practical ABCC4 inhibitors for clinical use, an important modification on current molecules should aim to overcome off-target effects by achieving tumor-specific targeting.

Second, if obstacles in the deployment of traditional ABCC4 inhibitors persist, alternative strategies to suppress ABCC4 expression, such as miRNA interference or monoclonal antibody therapy (mAb), could be more viable treatment options. Notably, the burgeoning field of Targeted Protein Degradation (TPD) (including PROTAC, LYTAC, AbTAC and AUTAC) has promise by targeting proteins of interest with a specific-binding ligand or bispecific antibody to activate the cells’ natural protein degradation networks [9]. The goal of these TPD strategies is complete degradation of the disease-causing protein to ablate its associated biological functions [9]. As ABCC4 is a membrane-bound protein, the use of LYTAC or AbTAC are preferred as they can target transmembrane proteins [9]. One significant hurdle for implementing this treatment on SHH-MB is producing specific-binding ligands or bispecific antibodies that cross the blood-brain barrier (BBB). Therefore, developing ABCC4-binding ligands or bispecific antibodies that can cross the BBB is needed before this strategy is viable for clinical use.

Finally, what is the best way to achieve maximal clinical efficacy of an ABCC4 inhibitor? Wijaya et al., reported a significant correlation between high expression of ABCC4 and poor prognosis in human SHH-MB suggesting that the extent of ABCC4 expression in SHH-MB could be used as a marker to determine disease severity. Therefore, when deciding whether to treat SHH-MB patients with an ABCC4 inhibitor, several parameters should be considered: 1) the level of ABCC4 expressed in the tumor. The higher the ABCC4 expression, the greater the need for treatment with an ABCC4 inhibitor; 2) the extent of ABCC4 suppression needed to improve patient outcome. Ideally, the inhibitor would efficiently block tumor growth but permit basic functions of ABCC4; and 3) the benefit of combination therapy with a chemotherapeutic that is an ABCC4 substrate (e.g., topotecan). With combination therapy, better tumoricidal activity is expected as inhibition of ABCC4 would increase intratumoral concentration of the other chemotherapeutic, however, dosages of both should be carefully selected to avoid unfavorable toxicity. Thus, we propose the establishment of a clinical workflow, from diagnosis and ABCC4 expression profiling to the decision of treatment and dosing, would be desirable to achieve favorable efficacy (Figure 1B).

The discovery of novel modulators of the SHH pathway to advance SHH-MB treatment is greatly needed. Use of a data-driven systems biology approach led to the identification ABCC4 as a novel target of SHH-MB. Presumably this method could be expanded to uncover ABCC4’s role in other diseases with altered SHH signaling or other cancers in which high expression of ABCC4 is a hallmark of poor prognosis (Figure 1B). It is likely that an ABCC4 inhibitor would be an attractive therapeutic approach in these diseases, but the challenges presented above need to be overcome. Therefore, future studies should aim to obtain a better understanding of ABCC4’s biological roles in different cancers and to overcome therapeutic barriers in implementation of ABCC4 inhibitors. These aims are fundamental in directing the development of ABCC4 inhibitors towards the ideal therapeutic strategy and providing a helpful guidance for clinical use to attain maximum efficacy.
